# Risk factors for voriconazole-associated hepatotoxicity in patients with liver dysfunction: a retrospective nested case–control study

**DOI:** 10.3389/fphar.2025.1625003

**Published:** 2025-08-26

**Authors:** Jing Ren, Xinfeng Cai, Wei Ge, Jinlin Guo, Shan Wang, Qinhui Wang, Linna Liu, Le Yang, Qi Yang

**Affiliations:** ^1^ Department of Pharmacy, Tangdu Hospital, Fourth Military Medical University, Xi’an, Shaanxi, China; ^2^ Department of Pharmacy, Shanxi Province Cancer Hospital, Taiyuan, Shanxi, China; ^3^ Department of Disease Prevention and Control, Tangdu Hospital, Fourth Military Medical University, Xi’an, Shaanxi, China; ^4^ Department of Pharmacy, Shanxi Provincial People’s Hospital, Taiyuan, Shanxi, China; ^5^ Department of Pharmacy, New York University, Langone Hospital–Long Island, Mineola, NY, United States

**Keywords:** voriconazole, hepatotoxicity, acute liver injury, therapeutic drug monitoring, trough concentration

## Abstract

**Introduction:**

Voriconazole is widely used to prevent and treat invasive aspergillosis. However, its use is restricted by adverse effects, including acute liver injury (ALI). Patients with hepatic insufficiency are often more susceptible to voriconazole-induced liver injury than those with normal hepatic function. The aim of this study was to determine the incidence and risk factors of ALI in patients with mild or moderate liver dysfunction during voriconazole treatment.

**Methods:**

This single-center nested case–control study involved adult patients treated with voriconazole for at least 3 days. The Child–Pugh score is now extensively utilized to assess liver damage. The hepatotoxicity of voriconazole was assessed in patients with mild or moderate hepatic insufficiency (Child–Pugh A or B). ALI cases were matched with controls based on age and Child–Pugh score. Basic characteristics were compared between patients who developed ALI and those who did not by performing univariate and multivariate conditional logistic regression analyses. The optimal cutoff condition was determined using a receiver operating characteristic curve.

**Results:**

A total of 140 patients (ALI: *n* = 44; control: *n* = 96) were enrolled. The incidence of voriconazole-induced ALI in patients with mild or moderate liver dysfunction was 30.6%. The univariate analysis revealed trough voriconazole plasma concentration (VPC), voriconazole treatment duration, activated partial thromboplastin time, and intensive care unit admission as variables for the final analysis. Voriconazole-induced ALI was independently associated with trough VPC (odds ratio [OR]: 1.592, *p* = 0.013) and voriconazole treatment duration (OR: 1.057, *p* = 0.005). Notably, the optimal cutoff for treatment duration was 10 days and the recommended trough VPC threshold was 3.81 mg/L.

**Conclusion:**

The incidence of voriconazole-induced ALI was higher in patients with mild or moderate liver dysfunction than in the general population. Trough VPC and voriconazole treatment duration are two independent risk factors of ALI. Therefore, voriconazole should be administered with caution to these patients. A lower target trough VPC (<3.81 mg/L) is recommended to minimize the risk of ALI in patients with mild-to-moderate liver dysfunction.

## 1 Introduction

Voriconazole is a triazole antifungal agent with high efficacy against most *Aspergillus* species (Scott and Simpson, 2007; Pascual et al., 2008). It is commonly used to prevent and treat invasive fungal infections and is recommended as first-line therapy for invasive aspergillosis (IA) (Patterson et al., 2016; Moriyama et al., 2017). However, its use is limited by adverse drug reactions, including visual disturbances, encephalopathy, rash, and liver injury (Levine and Chandrasekar, 2016; Benitez and Carver, 2019).

Voriconazole pharmacokinetics (PK) are complex, with high inter- and intra-individual variability (Purkins et al., 2002; Saleh et al., 2024). However, trough voriconazole plasma concentration (VPC) is correlated with efficacy and adverse events (Hu et al., 2025; Jin et al., 2016). Voriconazole is metabolized by hepatic cytochrome P450 (CYP) isoenzymes, primarily by CYP2C19 and partially by CYP2C9 and CYP3A4 (Hyland et al., 2003; Zonios et al., 2014). Trough VPC may be influenced by various confounding factors, including drug–drug interactions and CYP2C19 gene polymorphisms (Wang et al., 2014; Hu et al., 2023). Therefore, therapeutic drug monitoring (TDM) of trough VPC is recommended (Park et al., 2012; Takesue et al., 2022).

Voriconazole disposition is influenced by liver function, with reduced metabolic enzyme activity observed in patients with liver dysfunction (Tang et al., 2021). General hepatotoxicity from voriconazole use occurs in 69% of patients with liver dysfunction and in only 15.82% of the general population (Solís-Muñoz et al., 2013; Xiao et al., 2023; Hu et al., 2024). The incidence of ALI, which is a severe form of hepatotoxicity, in patients with mild-to-moderate liver dysfunction treated with voriconazole is unknown. These patients are often treated with other potentially hepatotoxic drugs and may have underlying conditions that predispose them to ALI. Thus, understanding the risk factors for voriconazole-induced ALI is essential for improving drug safety.

The aim of this nested case–control study was to: (1) determine the incidence of ALI in patients with mild-to-moderate liver dysfunction receiving voriconazole and (2) identify the risk factors of voriconazole-induced ALI in this population.

## 2 Materials and methods

### 2.1 Study design

This retrospective nested case–control study was conducted in Tangdu Hospital, Fourth Military Medical University, a teaching hospital with 3,200 beds. Patients admitted to the hospital between December 2022 and April 2024 were included in this study. The research was conducted in accordance with the tenets of the Declaration of Helsinki and approved by the Ethics Committee of Tangdu Hospital, Fourth Military Medical University, Shaanxi, China (approval no. K202504-70). Owing to the retrospective nature of the study, the requirement for informed consent from patients was waived by our Institutional Review Board.

### 2.2 Inclusion and exclusion criteria

The inclusion criteria were patients aged ≥18 years, with mild or moderate liver insufficiency, with no history of viral hepatitis, and with at least one measured trough VPC. Liver impairment was evaluated using the Child–Pugh classification. Initially developed to predict operative mortality in patients with liver cirrhosis, the Child–Pugh score is now extensively utilized to assess liver damage (Hu et al., 2024). The FDA recommends the use of the Child–Pugh scoring system for studies assessing hepatic dysfunction (Chang et al., 2013). Patients with Child–Pugh A are classified as having mild hepatic impairment and those with Child–Pugh B as having moderate hepatic insufficiency (Tang et al., 2021). Therefore, in our study, patients with mild or moderate hepatic impairment (Child–Pugh A or B) who received voriconazole for at least 3 days were included.

**FIGURE 1:**
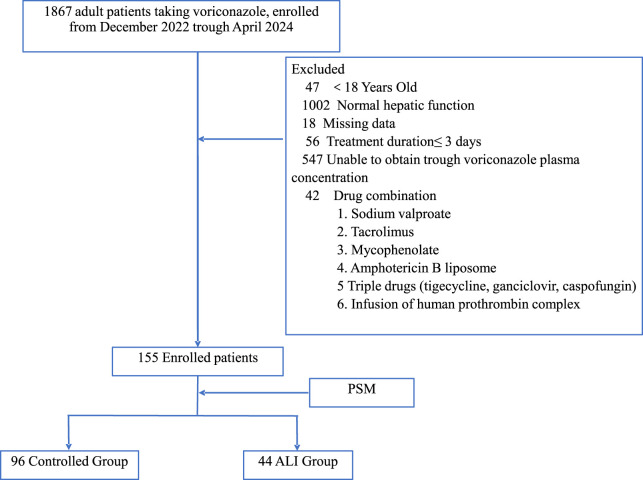
Patient inclusion protocol and nested case-controlled design. PSM, propensity score matching; ALI, acute liver injury.

The exclusion criteria were concurrent treatment with liposomal formulations; valproic acid; tacrolimus; mycophenolate mofetil; or a triple regimen of tigecycline, ganciclovir, and caspofungin (Figure 1). Patients with missing data were excluded from the study.

### 2.3 Definition of ALI

The reference ranges of parameters for normal liver function were as follows: total bilirubin (TBIL) level, 2–26 μmol/L; alanine aminotransferase (ALT) level, 9–50 U/L; and aspartate transaminase (AST) level, 15–40 U/L.

In individuals with normal liver function before voriconazole administration, ALI was diagnosed when the TBIL level exceeded twice the upper normal limit or if the ALT or AST level exceeded five times the upper normal limit after voriconazole treatment.

For individuals with abnormal liver function before voriconazole administration, ALI was defined as an increase in the TBIL, ALT, or AST level of more than twice the baseline value. Baseline values were measured ≤24 h before voriconazole initiation.

All patients diagnosed with ALI comprised the ALI group, whereas individuals who met the inclusion criteria without ALI formed the control group.

The causality of voriconazole hepatotoxicity was assessed using the Roussel Uclaf Causality Assessment Method (RUCAM) scale (Andrade et al., 2007).

### 2.4 Data collection

The hepatotoxicity of voriconazole was assessed in patients with mild or moderate hepatic insufficiency (Child–Pugh A or B). Five variables (encephalopathy, ascites, bilirubin, albumin [ALB], and prothrombin) were used to classify all patients into one of the following two groups: Child–Pugh A (mild hepatic impairment) and Child–Pugh B (moderate hepatic impairment). The medical records of each patient were reviewed using a data collection template, and the following data were extracted: demographic characteristics (age, sex, and body weight), laboratory data (AST, ALT, TBIL, ALB, and serum creatinine levels and prothrombin time), concomitant antibiotic medications (tigecycline), trough VPC, intensive care unit (ICU) admission (yes/no), underlying medical conditions, etiology, site of fungal infection, all-cause mortality rate, voriconazole treatment duration, and activated partial thromboplastin time (APTT).

### 2.5 Statistical analyses

Propensity score matching (PSM) (1:3) was used to adjust the baseline data (age and Child–Pugh score) of the ALI and control groups. The Shapiro–Wilk test was employed to determine whether the data were normally distributed. Continuous variables are presented as mean ± standard deviation or median and interquartile range (IQR), whereas categorical variables are expressed as number (%). The χ^2^ test or Fisher’s exact test was used to compare the categorical variables. Student’s t-test or Kruskal–Wallis H test was used to compare continuous variables. Data were modeled using a conditional logistic regression model; the dependent variable was ALI (yes/no) and the main exposure variables were treatment duration, trough VPC, ICU admission (yes/no), APTT, and drug combination (tigecycline). Conditional logistic regression analysis was used to calculate the odds ratio (OR) with 95% confidence interval (CIs) to evaluate the relationships between variables and ALI risk factors. Each variable was analyzed individually. Covariates with a *p*-value of <0.1 in the univariate analysis were entered into the multivariate analysis. For all final analyses, results with *p* < 0.05 were considered statistically significant.

Receiver operating characteristic (ROC) curve analysis was performed to explore the optimal cutoff point for VPC and ALI occurrence. All statistical analyses were conducted using xsmartanalysis (https://www.xsmartanalysis.com).

## 3 Results

### 3.1 Baseline patient characteristics

From December 2022 to April 2024, 155 patients with Child–Pugh score of A or B before initiating voriconazole treatment were included in the study. A significant difference in age was observed before PSM; however, this difference was no longer significant after PSM. After PSM, the control group comprised 96 patients and the ALI group comprised 44 patients (Figure 1). In the control group, 53 patients (55.2%) were male individuals and 76 patients (79.2%) were admitted to the ICU. In the ALI group, 26 patients (59.1%) were male individuals and 34 patients (77.3%) were admitted to the ICU. Additionally, 18 patients (18.8%) in the control group and 13 patients (29.5%) in the ALI group received tigecycline. Of the 140 patients included in the analysis, the lung was the most common fungal infection site, and immunosuppression (71 patients, 50.7%), leukemia (51 patients, 36.4%), and hypoproteinemia (60 patients, 42.9%) were the most prevalent underlying conditions (Table 1).

**TABLE 1:** Baseline information.

Variable	Control group (*n* = 96)	ALI group (*n* = 44)	*p*
Age (years), median [IQR]	59.0 [49.0–65.0]	61.0 [54.0–68.0]	0.174
Sex			
Male	53 (55.2%)	26 (59.1%)	0.667
Female	43 (44.8%)	18 (40.9%)	
ICU, *n* (%)			
Yes	76 (79.2%)	34 (77.3%)	0.800
No	20 (20.8%)	10 (22.7%)	
Drug combination, *n* (%)			
Tigecycline			0.153
Yes	18 (18.8%)	13 (29.5%)	
No	78 (81.3%)	31 (70.5)	
Underlying condition, *n* (%)			
Renal insufficiency	17 (17.7%)	10 (22.7%)	NaN
Immunosuppressive state	45 (46.9%)	26 (59.1%)	
Leukemia	41 (42.7%)	10 (22.7%)	
Hypoproteinemia	32 (33.3%)	28 (63.6%)	
COPD	4 (4.2%)	1 (2.3%)	
Other	8 (8.3%)	1 (2.3%)	
Site of fungal infection, *n* (%)			
Unclear	46 (47.9%)	32 (72.7%)	NaN
Blood	16 (16.7%)	6 (13.6%)	
Lung	32 (33.3%)	4 (9.1%)	
Intracranial	1 (1.0%)	1 (2.3%)	
Other (urinary, oral, or abdominal)	6 (6.3%)	3 (6.8%)	
All-cause mortality rate	10 (10.4%)	13 (29.5%)	0.005
Trough concentration (mg/L), median [IQR]	2.700 [1.460–3.740]	5.070 [4.050–5.960]	<0.001
Length of treatment (days), median [IQR]	9.000 [7.000–16.000]	21.000 [13.000–28.000]	<0.001
Cr (μmol/L), median [IQR]	55.800 [46.000–67.900]	67.700 [49.200–89.300]	0.128
Child–Pugh Score, median [IQR]	5.000 [5.000–6.000]	6.000 [5.000–6.000]	0.126

ALI, acute liver injury; COPD, chronic obstructive pulmonary disease; ICU, intensive care unit; IQR, interquartile range; ALI, acute liver injury; Cr, creatinine; NaN, not a number.

### 3.2 ALI

ALI was observed in 44 (31.4%) of the 140 patients. The rate of tigecycline use was higher in the ALI group than in the control group, although the difference was not significant between the groups (*p* > 0.05). Drug causality was assessed using the RUCAM scale. Among these 44 patients, all scored above six points on the RUCAM scale, indicating a high probability for drug-related hepatotoxicity. The proportion of individuals with hypoproteinemia in the ALI group was 63.6%, which was higher than that in the controlled group (33.3%). The patients in our study could have had leukemia and hypoalbuminemia or renal insufficiency and hypoalbuminemia simultaneously. Therefore, we did not include underlying conditions as a separate variable in the statistical analysis. The median voriconazole treatment duration was 21 (IQR: 13–28) days in the ALI group and 9 (IQR: 7–16) days in the control group. The univariate analysis revealed trough VPC, voriconazole treatment duration, APTT, and ICU admission as potentially significant variables; they were input into the multivariate regression analysis. The rate of tigecycline combination use was higher in the ALI group than in the control group (29.5% vs. 18.8%, *p* = 0.153), though the difference was not significant. Ultimately, trough VPC (OR: 1.59 [1.29–2.02], *p* = 0.013) and voriconazole treatment duration (OR: 1.06 [1.03–1.10], *p* = 0.005) were identified as independent risk factors for hepatotoxicity. ICU admission was not significantly associated with ALI (*p* = 0.881), and so was APTT (*p* = 0.155) (Table 2). Furthermore, 10 (10.4%) patients in the control group and 13 (29.5%) in the ALI group died during voriconazole therapy. The all-cause mortality rate was significantly higher in the ALI group than in the control group (*p* = 0.005). The majority of these fatalities were mostly attributable to progression of their underlying hematologic malignancies rather than other causes.

**TABLE 2:** Results of conditional logistic regression for the ALI and control groups.

Risk factor	Univariate regression analysis		Multivariate regression analysis	
	Odds ratio (95% CI)	*P*	Odds ratio (95% CI)	*P*
Trough VPC	1.15 (1.06–1.31)	0.017	1.59 (1.29–2.02)	0.013
Duration of voriconazole treatment	1.05 (1.02–1.08)	0.001	1.06 (1.03–1.10)	0.005
APTT	1.03 (0.99–1.06)	0.08	1.03 (0.99–1.07)	0.155
ICU admission	1.13 (0.87–2.6)	0.086	0.92 (0.31–2.59)	0.881

ALI, acute liver injury; APTT, activated partial thromboplastin time; ICU, intensive care unit.

### 3.3 Evaluation of the optimal trough voriconazole concentration and voriconazole treatment duration

**FIGURE 2:**
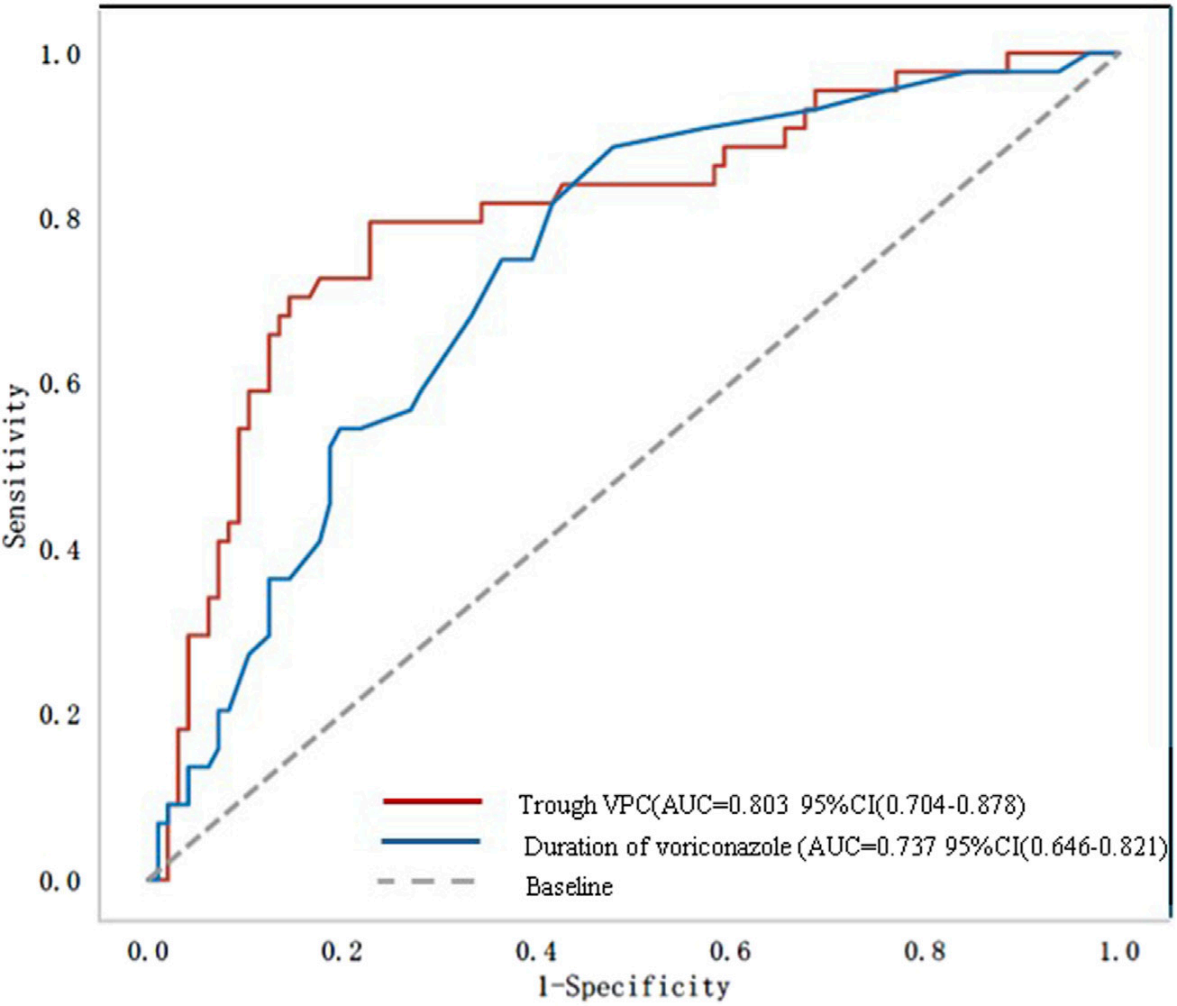
Receiver operating characteristic (ROC) curve. AUC, area under the curve; VPC, voriconazole plasma concentration.

Figure 2 shows the ROC curve related to the incidence of ALI. The area under the curve (AUC) was 0.81 (95% CI: 0.70–0.88) for trough VPC and 0.74 (95% CI: 0.65–0.82) for voriconazole treatment duration. The optimal cutoffs were 10 days for treatment duration and 3.81 mg/L for trough VPC (Table 3).

**TABLE 3:** Receiver operating characteristic (ROC) curve analysis.

Variable	AUC	Sensitivity	Specificity	Cutoff
Trough VPC	0.81 (0.70–0.88)	0.79 (0.59–0.89)	0.77 (0.71–0.92)	3.810
Duration of voriconazole treatment	0.74 (0.65–0.82)	0.87 (0.55–0.97)	0.52 (0.46–0.86)	10.000

AUC, area under curve; VPC, voriconazole plasma concentration.

## 4 Discussion

Voriconazole is the standard therapy for IA. In addition to that of immunosuppression, the incidence of aspergillosis appears to be increasing in patients outside commonly recognized risk groups, including those with chronic obstructive pulmonary disease (COPD) (Solís-Muñoz et al., 2013). In the present study, hypoproteinemia and renal insufficiency accounted for a large proportion of cases. The primary reason for initiating empiric therapy was suspected IA of the lungs.

Voriconazole is effective against most *Aspergillus* species, and it reaches high concentrations in the lung tissue. However, frequent adverse effects, such as hepatotoxicity, limit its use. Although the incidence of voriconazole-associated hepatotoxicity is moderate in the general public (Levine and Chandrasekar, 2016), it is more common in patients with liver dysfunction (Hu et al., 2024). In the present study, we evaluated voriconazole-induced ALI, specifically in patients with mild or moderate hepatic dysfunction. Among patients with liver dysfunction (Child–Pugh A or B), 44 (30.6%) developed ALI during voriconazole therapy. Therefore, we sought to identify clinical risk factors to reduce ALI incidence in this population.

Previous research has focused on the safety of voriconazole in patients with severe liver dysfunction (Solís-Muñoz et al., 2013). However, studies on the hepatotoxicity of voriconazole in patients with mild-to-moderate liver dysfunction are lacking. Our study demonstrated that patients with liver dysfunction are at a high risk of developing voriconazole-induced ALI. High trough VPC was identified as an independent risk factor for voriconazole-associated hepatotoxicity. The optimal steady trough concentration for voriconazole, per Infectious Disease Society of America guidelines, is 1.0–5.5 mg/L (Patterson et al., 2016). Similarly, a population PK study in patients with liver dysfunction reported an association between hepatotoxicity and trough VPC levels of <5.1 mg/L. Meanwhile, in the present study, the optimal trough VPC cutoff to prevent ALI was 3.81 mg/L, suggesting that trough VPC levels of >3.81 mg/L in patients with liver dysfunction (Child–Pugh A or B) receiving voriconazole pose a higher risk of ALI. The potential underlying mechanisms are as follows. (1) Voriconazole is primarily metabolized by CYP450. Hepatic impairment decreases CYP450 enzyme activity, leading to accumulation of the parent drug in the liver, which may cause liver injury (Hu et al., 2024). (2) Decreased bile excretion may lead to the retention of drugs or their toxic metabolites (such as voriconazole N-oxide) in the liver (Yuan and Kaplowitz, 2009). (3) Patients with hepatic impairment have insufficient glutathione reserves, impairing their ability to effectively detoxify reactive metabolites (European Association for the Study of the Liver, 2024). The correlation between trough VPC levels and hepatotoxicity highlights the significance of TDM for voriconazole, particularly in patients with liver dysfunction. Adjusting the voriconazole dosage based on trough VPC levels could reduce the risk of ALI without necessitating drug discontinuation for drug-induced hepatotoxicity. The duration of voriconazole treatment is another independent risk factor for hepatotoxicity. Our study demonstrated a significantly increased risk of ALI associated with voriconazole treatment duration exceeding 10 days in patients with liver impairment. Clinicians should monitor liver function in patients receiving voriconazole therapy for more than 10 days. For patients anticipated to require voriconazole therapy for more than 10 days, proactive assessment to minimize other hepatotoxic exposures (e.g., sodium valproate, paracetamol, and macrolide antibiotics) is necessary. Our findings support targeting a trough concentration of <3.81 mg/L for patients with hepatic dysfunction to reduce the incidence of ALI. A trough level of ≥1.0 mg/L is strongly recommended to improve efficacy. Trough levels of ≥2.0 mg/L are suggested for invasive aspergillosis (Japanese Antimicrobial Therapeutic Drug Monitoring Guideline Committee, 2022). If voriconazole trough levels approach 3.81 mg/L, clinicians should monitor liver function more closely. If voriconazole trough levels are within 3.81–10 mg/L, the maintenance dosage of voriconazole is recommended to be decreased by 20%. If the trough VPC is above 10 mg/L, voriconazole administration is suggested to be skipped once, with the maintenance dosage decreased by 50%, followed by dosage adjustment based on the blood concentration (Chen et al., 2018). Considering that tigecycline can independently cause liver toxicity (Jiang et al., 2022), we hypothesized that its concomitant use with voriconazole may contribute to ALI. Accordingly, we included tigecycline use as a variable to assess its relationship with voriconazole-induced ALI. The rate of tigecycline use was higher in the ALI group than in the control group, although the difference was not significant between the groups (*p* > 0.05).

Certain underlying disease may influence the adverse effects of voriconazole. In a previous study, the risk of liver injury increased in patients with hypoproteinemia (Hirata et al., 2019). In the present study, we systematically collected and analyzed underlying medical conditions in patients, including renal insufficiency, immunosuppressive state, leukemia, hypoproteinemia, and COPD. As patients can suffer from multiple underlying diseases simultaneously. We did not include underlying medical condition as a separate variable in the statistical analysis. Hypoalbuminemia can increase the concentration of plasma-free voriconazole, distributing more free molecules to the liver and other tissues and organs, increasing the risk of adverse reactions.

This study had some limitations. First, only four variables were included in the multivariable analysis, all of which appeared significant (*p* < 0.1). A larger sample size could enhance the statistical power to identify additional risk factors in patients with liver dysfunction. Second, the diagnostic criteria for drug-induced ALI were inadequate. Although a temporal relationship exists between elevated liver enzyme levels and voriconazole use, biochemical abnormalities during drug administration may not accurately confirm drug-induced ALI. Third, serum voriconazole concentrations can vary in patients with different CYP2C19 genotypes, and this may influence drug safety (Wang et al., 2016). In addition, *CYP2C19* polymorphisms vary considerably between races. CYP2C19-poor metabolizer prevalence in China is considerably higher than that in Europe (specifically, Caucasians) and Africa. The applicability of our proposed trough VPC cutoff and hepatotoxicity risk predictions may be influenced by ethnicity-dependent genetic polymorphisms. In our study, the majority of patients did not undergo genetic polymorphism testing. Therefore, we did not incorporate this risk factor for voriconazole-induced ALI. Pharmacogenetic testing should be considered as a future direction to refine risk stratification and dosing. Finally, our study reflects the clinical context of a tertiary center in China. Regional variations in liver disease etiology may limit generalizability; for instance, alcoholic liver disease and metabolic dysfunction-associated steatotic liver disease prevail in Western populations. Future validation in multi-regional patients would strengthen voriconazole applicability.

This study has significant clinical implications for the management of patients with liver dysfunction undergoing voriconazole treatment. Trough VPC was identified as an independent risk factor for ALI, with a toxic threshold of 3.81 mg/L for patients with mild or moderate liver dysfunction. Therefore, TDM of voriconazole is crucial for this patient population to prevent discontinuation owing to ALI. A lower target trough VPC (<3.81 mg/L) is suggested for these patients. If the clinical response is inadequate at lower trough VPC levels, clinicians may consider alternative antifungals, such as caspofungin alone or in combination with nebulized amphotericin B.

Overall, this study revealed that voriconazole-induced ALI occurs in 30.6% of patients with mild-to-moderate liver dysfunction (Child–Pugh A and B). Independent risk factors for ALI include a trough VPC of >3.81 mg/L and a treatment duration exceeding 10 days. Further large-scale prospective studies are required to validate these risk factors and enhance voriconazole management in this population.

## Data Availability

The original contributions presented in the study are included in the article/supplementary material, further inquiries can be directed to the corresponding authors.

## Abbreviations

IA, invasive aspergillosis.
